# Modelling modal gating of ion channels with hierarchical Markov models

**DOI:** 10.1098/rspa.2016.0122

**Published:** 2016-08

**Authors:** Ivo Siekmann, Mark Fackrell, Edmund J. Crampin, Peter Taylor

**Affiliations:** 1 Systems Biology Laboratory, Melbourne School of Engineering, University of Melbourne, Melbourne, Australia; 2 Centre for Systems Genomics, University of Melbourne, Melbourne, Australia; 3 School of Mathematics and Statistics, University of Melbourne, Melbourne, Australia; 4 School of Medicine, University of Melbourne, Melbourne, Australia; 5 Australian Research Council Centre of Excellence in Convergent Bio-Nano Science and Technology, Melbourne, Australia; 6 Australian Research Council Centre of Excellence for Mathematical and Statistical Frontiers, Melbourne, Australia

**Keywords:** ion channels, modal gating, continuous-time hierarchical Markov model, inositol-trisphosphate receptor

## Abstract

Many ion channels spontaneously switch between different levels of activity. Although this behaviour known as modal gating has been observed for a long time it is currently not well understood. Despite the fact that appropriately representing activity changes is essential for accurately capturing time course data from ion channels, systematic approaches for modelling modal gating are currently not available. In this paper, we develop a modular approach for building such a model in an iterative process. First, stochastic switching between modes and stochastic opening and closing within modes are represented in separate aggregated Markov models. Second, the continuous-time hierarchical Markov model, a new modelling framework proposed here, then enables us to combine these components so that in the integrated model both mode switching as well as the kinetics within modes are appropriately represented. A mathematical analysis reveals that the behaviour of the hierarchical Markov model naturally depends on the properties of its components. We also demonstrate how a hierarchical Markov model can be parametrized using experimental data and show that it provides a better representation than a previous model of the same dataset. Because evidence is increasing that modal gating reflects underlying molecular properties of the channel protein, it is likely that biophysical processes are better captured by our new approach than in earlier models.

## 1. Introduction

Ion channels regulate the flow of ions across the cell membrane by stochastic opening and closing. As soon as it became possible to detect currents generated by the movement of charged ions through the channel via the patch-clamp technique [1], Colquhoun & Hawkes [2] developed the theory of modelling single ion channels with continuous-time Markov models which describe the time-course of opening and closing that is reflected in single-channel currents by stochastic jumps between zero (closed) and one or more small non-zero current levels in the pA range (open). The activity of an ion channel is usually measured by its open probability *P*_*O*_. But by 1979, Patlak *et al.* [3] had already observed spontaneous changes of channel activity in glutamate-activated channels, shortly afterwards followed by Magleby & Pallotta [4,5], who made similar observations in the calcium-activated potassium channel. Since then this phenomenon, known as modal gating, has been ubiquitously observed across a wide range of ion channels but the significance of modal gating has remained unclear. See Siekmann *et al.* [6] for a more comprehensive review of the experimental literature. Colquhoun & Hawkes [7] modified their general theory from Colquhoun & Hawkes [2] for the analysis of bursts. Bursts are defined as ‘closely spaced openings, separated by longer shut periods’ [7, p. 4], which means that they are related to modes with a high level of activity. Thus, the papers Colquhoun & Hawkes [2,7] contain a comprehensive theory for calculating various statistical properties of the channel kinetics from a given Markov model. However, the problem of constructing models that capture spontaneous changes of channel activity in a systematic way has, so far, not been addressed in the literature.

**Figure 1.**
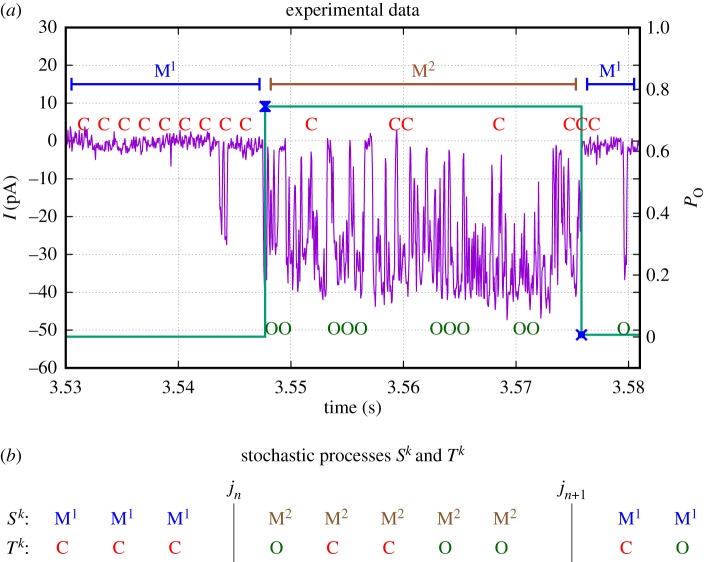
After a statistical analysis of modal gating [6], experimental data are partitioned into segments based on different levels of open probability *P*_*O*_ by inferring changepoints *j*_*n*_. For the small section of data shown in (*a*), the channel spontaneously jumps at *t*≈3.55 s from a low *P*_*O*_ close to zero (M^1^) to a high level of activity with *P*_*O*_≈75% (M^2^). At *t*≈3.575 s, the channel leaves the highly active mode M^2^ and returns to the low level of activity characteristic for M^1^. Through this segmentation, the original stochastic process *T*^*k*^ of open (O) and closed (C) events has been augmented by the additional information *S*^*k*^ of the mode (*M*^1^,*M*^2^… ) that the channel is in for a given point in time. The two coupled stochastic processes *S*^*k*^ and *T*^*k*^ will be represented by the continuous-time hierarchical Markov model developed in this study. (*a*) Experimental data and (*b*) stochastic processes *S*^*k*^ and *T*^*k*^.

In this study, we present a general framework for building data-driven models of ion channels that account for modal gating. This is essential for accurately representing the dynamics of an ion channel—instead of producing a misleading constant intermediate open probability *P*_*O*_, a model should represent the switching between highly different levels of activity characteristic of each mode. This is illustrated in figure 1 where data points labelled M^1^ form a segment characterized by a low open probability, whereas the segment labelled M^2^ is characterized by a high open probability. In a realistic time series, the changes between M^1^ and M^2^ occur on a time scale so slow that directly fitting a model (even if they have a sufficient number of open and closed states) to the data will not be able to resolve the infrequent switching between high and low open probabilities but instead will most likely lead to a model with a constant intermediate open probability. Moreover, modes of an ion channel have been associated with biophysical properties of the channel protein [6]. Therefore, a model accounting for modal gating is more likely to appropriately relate the dynamics of ion channels to underlying biophysical states of the channel protein.

Blatz & Magleby [8] presented an early modelling study of three modes observed in a chloride channel. They chose segments representative of an inactive, an active and a flicker mode and went through a thorough model selection process. In this way, they obtained models for each of the three modes. They estimated the order of magnitude of the transitions between these modes and presented a qualitative model structure that illustrates the transitions between the three modes. The model that will be developed here can be regarded as a quantitative development of the idea by Blatz & Magleby [8].

After this early study, modal gating has only rarely been considered in ion channel models. But recently, shortly after the discovery of modal gating in the inositol-trisphosphate receptor (IP_3_R) by Ionescu *et al.* [9]—an observation that has been received with great interest in the IP_3_R community, Mak & Foskett [10]—Ullah *et al.* [11] and Siekmann *et al.* [12] independently proposed two different models that represent modal gating in the IP_3_R. Both models are discussed in more detail in §5. The model by Ullah *et al.* [11] has most recently been used for investigating the influence of modal gating on calcium puffs [13] and for studying the impact of increased IP_3_R activity in Alzheimer's disease [14]. One difficulty in appropriately representing modal gating of ion channels in a model is the fact that for a time series of measurements collected from an ion channel, it is impossible to infer directly in which mode the channel is at a given point in time. However, Siekmann *et al.* [6] have shown how this information can be obtained by statistical changepoint analysis (figure 1). Previously, segments representative of different modes were either selected by visual inspection or by estimating the open probability using moving averages. Ionescu *et al.* [9] presented a heuristic algorithm that segments the data based on an analysis of burst durations and burst-terminating gaps. Siekmann *et al.* [6] detected mode changes by identifying significant changes of the open probability between adjacent segments in a time series recorded from an ion channel. In contrast with previous approaches, the uncertainty of the inferred changepoints where mode switching has supposedly occurred can be comprehensively assessed because Siekmann *et al.* [6] calculated probability distributions for the changepoint locations.

**Figure 2.**
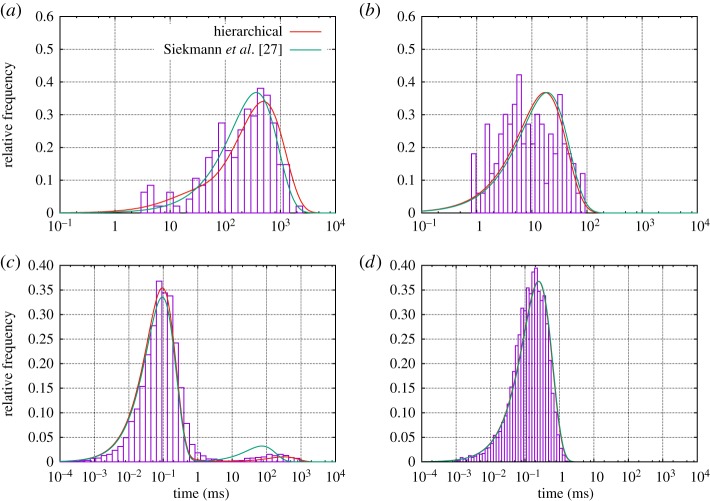
The model from Siekmann *et al.* [12] and the new hierarchical model are compared for a dataset from type I IP_3_R for 10 μM IP_3_, 5 mM ATP and 0.01 μM Ca^2+^.(*a*) The fit of the new model to the empirical sojourn time density in mode M^1^ (shown in red)is slightly improved in comparison with the original model (shown in green). This improved fit of the modal kinetics clearly improves the fit to the closed time densities shown in (*c*). (*a*) Sojourn time density in M^1^, (*b*) sojourn time density in M^2^, (*c*) closed time density and (*d*) open time density.

**Figure 3.**
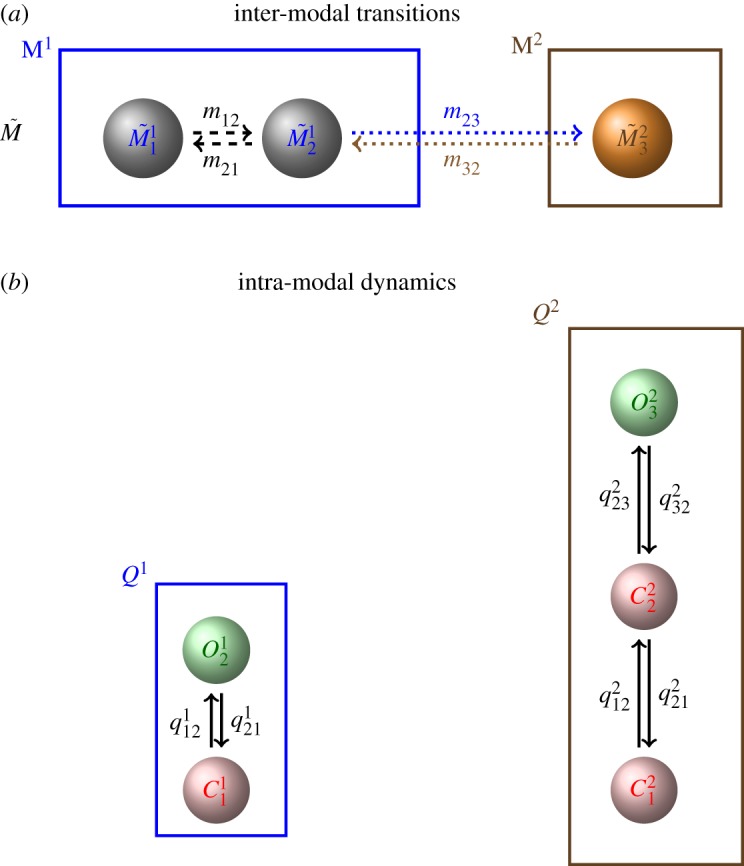
Modular components of a model for modal gating. (*a*) An example for an aggregated Markov model $\tilde{M}$ representing inter-modal dynamics, the stochastic switching between two modes, M^1^ and M^2^. M^1^ is modelled by an aggregate of two states, whereas M^2^ is represented by one state. The rates *m*_23_ and *m*_32_ stand for transitions between both modes. Note that $\tilde{M}$ may, in general, represent transitions between more than two modes, therefore, the states ${\tilde{M}}_{j}^{i}$ are numbered consecutively by subscripts *j*, whereas the superscripts *i* indicate the mode *M*^*i*^. (*b*) Models *Q*^1^ and *Q*^2^ representing the stochastic opening and closing that is characteristic of mode M^1^ or M^2^, respectively. The states $C_{k}^{i}$ and $O_{k}^{i}$ are numbered similar to the ${\tilde{M}}_{j}^{i}$. Note that *k*=1,…,*n*_*i*_ for each mode *M*^*i*^ in contrast with the states ${\tilde{M}}_{j}^{i}$ where the index *j* runs from 1 to the total number of states. In figure 4, we show how $\tilde{M}$ and the *Q*^*i*^s are combined in a model that accurately represents both inter-modal transitions as well as intra-modal kinetics. (*a*) Inter-modal transitions and (*b*) intra-modal dynamics.

As a result, after this analysis has been carried out, for each point in the time series it is not only known if the channel is open (O) or closed (C), but also—with an associated level of uncertainty calculated by the method—in which of the modes *M*^1^,*M*^2^,… the channel is. Previously, we observed stochastic switching between a nearly inactive mode M^1^ and a highly active mode M^2^ in data from the IP_3_R [6]. In this paper, we will represent the stochastic process of switching between an arbitrary number of different modes *M*^*i*^ by a continuous-time Markov model with infinitesimal generator $\tilde{M}$. For data by Wagner & Yule [15], empirical histograms suggest that the sojourn time distribution $f_{M^{1}}(t)$ within mode M^1^ is not exponential (see figs 5 and 6 in Siekmann *et al.* [6] and figures 2*a* and 5). For this reason, in general, more than one state is needed for accurately representing the process of switching between modes. This means that modal sojourn times are represented by phase-type distributions, a class of distributions which is defined by the time a Markov chain spends in a set of transient states until exiting to an absorbing state [16,17]. We assume that the infinitesimal generator $\tilde{M}$ representing the switching between modes *M*^*i*^, *i*=1,… *n*_*M*_, has the following block structure:

1.1 M~=(M~1,1|M~1,2|…|M~1,nMM~2,1AA|M~2,2|…|M~2,nM⋮⋱⋮⋮⋱⋮⋮⋱⋮M~nM,1|………|M~nM,nM),

where the block matrices ${\tilde{M}}^{i,i}\in R^{m_{i}\times m_{i}}$, $m_{i}\in N$, on the diagonal describe transitions between states that represent the same mode *M*^*i*^, whereas the off-diagonal blocks ${\tilde{M}}^{i,j}\in R^{m_{i}\times m_{j}}$ represent transitions between states representing different modes *M*^*i*^ and *M*^*j*^, *i*≠*j*. An example for a model for switching between two modes M^1^ and M^2^ is shown in figure 3*a*.

Our modal gating analysis illustrated in figure 1 not only enables us to represent the stochastic process of switching *between* modes *M*^*i*^ but by studying the dynamics within representative segments we can investigate the processes of stochastic opening and closing characteristic of each mode. For the example in figure 1, the dynamics *within* mode M^2^ can be analysed by considering the sequence of open and closed events between *j*_*k*_ and *j*_*k*+1_. The dynamics within a mode *M*^*i*^ can be represented by a Markov model with infinitesimal generator *Q*^*i*^ which is obtained by fitting to representative segments of the same mode [12]. Similar to the sojourn times in the modes *M*^*i*^, the open and closed time distributions *f*_*O*_(*t*) and *f*_*C*_(*t*), respectively, are non-exponential and more than one open or closed state may be needed for accurately representing the dynamics. For the example shown in figure 1, we obtain two models with infinitesimal generators *Q*^1^ and *Q*^2^ (figure 3*b*).

In this paper, we develop a new mathematical model, the continuous-time hierarchical Markov model, that accounts simultaneously for both transitions *between* modes as well as the stochastic opening and closing *within* modes. A hierarchical Markov model in discrete time has been previously described by Fine *et al.* [18] but because we are not aware of a continuous-time version discussed in the literature, we develop the mathematical theory in detail and prove some fundamental properties. For ion channel modelling a continuous-time representation of the dynamics is more appropriate because it is commonly assumed that ion channels are able to make faster transitions than currently resolved by experiments. For the example of modal gating, we assume that switching between modes *M*^*i*^ is a top-level process that regulates the bottom-level process, the opening and closing of the channel characteristic of a particular mode *M*^*i*^. This is illustrated in figure 3.

The states ${\tilde{M}}_{j}^{i}$ are numbered consecutively by subscripts *j*, whereas the superscripts *i* indicate the mode *M*^*i*^. While the model is in mode M^1^ or analogously within one of the states ${\tilde{M}}_{1}^{1}$ or ${\tilde{M}}_{2}^{1}$ (figure 3*a*), its opening and closing is described by the infinitesimal generator *Q*^1^ (figure 3*b*). As soon as M^1^ is left to state ${\tilde{M}}_{3}^{2}$, the current state of model *Q*^1^ is vacated and a state of model *Q*^2^ is entered. Now, opening and closing is accounted for by *Q*^2^ until the state ${\tilde{M}}_{3}^{2}$ and mode M^2^ is left and state ${\tilde{M}}_{2}^{1}$ is entered.

**Figure 4.**
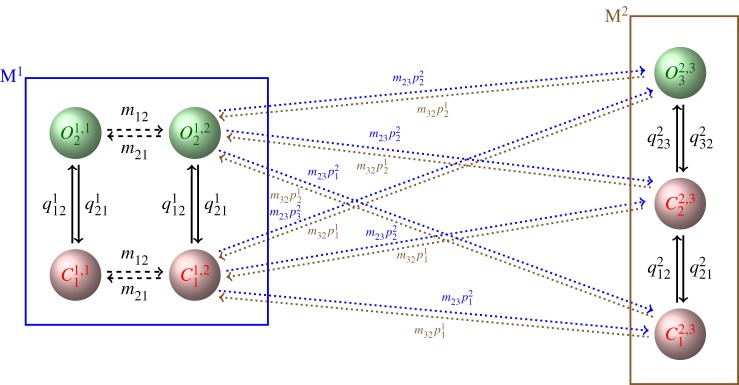
Aggregated Markov model that represents both transitions between modes M^1^ and M^2^ according to model $\tilde{M}$ (figure 3*a*) as well as stochastic opening and closing consistent with models *Q*^1^ and *Q*^2^ (figure 3*b*). The open and closed states are $O_{k}^{i,j}$ and $C_{k}^{i,j}$, respectively, where the superscripts *i*,*j* refer to the state ${\tilde{M}}_{j}^{i}$ in the model shown in figure 3*a*, whereas the subscript *k* is the index of the state within a model *Q*^*i*^ shown in figure 3*b*. This illustrates that the state set of the full model is obtained by the Cartesian product of states representing the modes *M*^*i*^ with the states of the model *Q*^*i*^. Owing to the transitions *m*_12_ and *m*_21_ between the two states representing M^1^, in the full model there are two copies of model *Q*^1^ connected by transition rates *m*_12_ and *m*_21_. For transitions between modes it is decided stochastically in which state the target mode is entered. The transitions are determined by initial distributions over the states of the models *Q*^*i*^. Thus, for our example, we have to choose two stochastic vectors $p^{1}=(p_{1}^{1},p_{2}^{1})$ and $p^{2}=(p_{1}^{2},p_{2}^{2},p_{3}^{2})$ that give the initial distributions over the states of *Q*^1^ and *Q*^2^. In order to ensure that the states are indeed entered with the chosen initial distribution, the rates *m*_23_ exiting M^1^ and *m*_32_ exiting M^2^ are weighted with *p*^1^ and *p*^2^.

The transitions between modes described via $\tilde{M}$ and the dynamics within modes captured by *Q*^*i*^ illustrated in figure 3 can be represented in a Markov model with infinitesimal generator *M* that is derived from the individual components $\tilde{M}$ and *Q*^*i*^. The idea is illustrated in figure 4 and developed formally in §2.

In order to account for the states ${\tilde{M}}_{j}^{i}$ as well as the states $O_{k}^{i}$ and $C_{k}^{i}$ representing the opening and closing within *M*^*i*^, the state space of the full model consists of the Cartesian products of the ${\tilde{M}}_{j}^{i}$ with the $O_{k}^{i}$ and $C_{k}^{i}$. Thus, the state space of the full model consists of open and closed states $O_{k}^{i,j}$ and $C_{k}^{i,j}$, respectively, where the superscripts *i*,*j* refer to the state ${\tilde{M}}_{j}^{i}$ in the model shown in figure 3*a*, whereas the subscript *k* is the index of the state within a model *Q*^*i*^ shown in figure 3*b*. For the example shown in the figure, the closed states $C_{1}^{1,1}$ and $C_{1}^{1,2}$ as well as the open states $O_{2}^{1,1}$ and $O_{2}^{1,2}$ are connected by the transition rates *m*_12_ and *m*_21_. Because M^1^ is modelled by two states ${\tilde{M}}_{1}^{1}$ and ${\tilde{M}}_{2}^{1}$, two ‘copies’ of *Q*^1^ appear in the full model, whereas there is only one ‘copy’ of *Q*^2^ which is represented by only one state in $\tilde{M}$.

For transitions between modes, it is decided stochastically in which state the target mode is entered. The transitions are determined by initial distributions over the states of the models *Q*^*i*^. Thus, for our example, we have to choose two stochastic vectors $p^{1}=(p_{1}^{1},p_{2}^{1})$ and $p^{2}=(p_{1}^{2},p_{2}^{2},p_{3}^{2})$ that give the initial distributions over the states of *Q*^1^ and *Q*^2^, respectively. For simplicity, we assume that this initial distribution does not depend on the state from which the transition originates so that—independent of the originating state—each state in the target model is entered with the same probability. In order to ensure that the states of *Q*^*i*^ are indeed entered with the chosen initial distribution, the transition rates have to be ‘split’ accordingly. For our example, the rates *m*_23_ exiting M^1^ and *m*_32_ exiting M^2^ are weighted with the stochastic vectors *p*^1^ and *p*^2^. The mathematical details of the construction of this model are presented in §2.

It is a strength of our approach that it enables us to build data-driven models of modal gating in a modular way. After segmenting ion channel data with the method by Siekmann *et al.* [6], we obtain a stochastic sequence of events *M*^*i*^ that describes the time course of transitions between different modes. The infinitesimal generators $\tilde{M}$ and the *Q*^*i*^ can then be parametrized from these data. We demonstrate the practical implementation of this approach in §3 using experimental data by Wagner & Yule [15] and compare the results with our previously published model of the same dataset [12].

We investigate the mathematical structure of the continuous-time hierarchical Markov model in more detail in §4. In particular, we show that many important properties of the infinitesimal generator *M* of the full model can be derived from the generators $\tilde{M}$ and *Q*^*i*^. We expect that similar to its discrete-time counterpart [18], the continuous-time hierarchical Markov model will have a variety of applications beyond the modelling of modal gating considered here.

We discuss our approach to modal gating in §5. In particular, we explain why our new modelling framework provides a representation of ion channel dynamics that is likely to provide a structure that realistically captures biophysical processes.

## 2. Material and methods

### (a) Preliminaries

We now develop formally the hierarchical Markov model illustrated graphically in figures 3 and 4. First, let us describe the structure of the probability distribution *p* over the states of the hierarchical Markov model. Let $v=(v^{1};v^{2};\ldots ;v^{n_{M}})$ denote a state probability distribution of the model $\tilde{M}$. That is, for *i*=1,…,*n*_*M*_, *v*^*i*^ is the probability distribution of the states in mode *M*^*i*^. In general, we will allow $\tilde{M}$ to be an aggregated Markov model so that each of the components *v*^*i*^ of the vector *v* may itself be a vector. With the term aggregated Markov model, we refer to a model where possibly multiple rather than one Markov states are used for representing the same experimental observation. Multiple states of the same aggregate cannot be directly distinguished based on experimental observations. Aggregated Markov models are capable of accounting for observations whose dwell times are distributed according to a mixture of exponentials rather than the exponentially distributed sojourns of single Markov states. We make the convention that components *v*^*i*^ and *v*^*j*^ that are meant to refer to a vector are separated by semicolons, whereas components of a vector are separated by commas. Let us first assume for simplicity that all modes *M*^*i*^ are represented by only one state so that the components *v*^*i*^ are scalars. Then the distribution *p* over the states of the full model *M* is a weighting of the distributions *w*^*i*^ over the distributions over the states of the models *Q*^*i*^. Thus, we obtain $p:=(v^{1}\cdot w^{1};\ldots ;v^{i}\cdot w^{i};\ldots ;v^{n_{M}}\cdot w^{n_{M}})$. Here ‘⋅’ denotes scalar multiplication of vectors *w*^*i*^ with scalars *v*^*i*^. If more than one state is needed for representing the modes *M*^*i*^, we must generalize appropriately the ‘weighting’ of a vector *w*^*i*^ with a vector *v*^*i*^. Such a generalization is provided by the tensor product ‘⊗’.

**Definition 2.1 (Kronecker product ⊗)**

We will only need the special case of the tensor product for matrices, the Kronecker product. Let $A\in R^{m\times n}$, $B\in R^{p\times r}$. Then

2.1 $$A⊗B:={\left(a_{ij}\cdot B\right)}_{1\leq i\leq m,1\leq j\leq n}=\left(\begin{array}{ccc} a_{11}B & \ldots & a_{1n}B\\ \vdots & \ddots & \vdots \\ a_{m1}B & \ldots & a_{mn}B \end{array}\right)\in R^{mp\times nr}.$$

The Kronecker product also applies to vectors by identifying column vectors with (*m*×1)- and row vectors with (1×*m*)-matrices.

**Definition 2.2 (Kronecker sum ⊕)**

The Kronecker sum of square matrices $A\in R^{m\times m}$ and $B\in R^{n\times n}$ is

2.2 $$A⊕B:=A⊗{\mathrm{id}}_{n}+{\mathrm{id}}_{m}⊗B\in R^{mn\times mn},$$

where *id*_*m*_ and *id*_*n*_ are the identity matrices of the respective dimensions.

For some properties of Kronecker product and sum that we require for our analysis of the hierarchical Markov model (§4), we refer to appendix A. For a distribution *v* over the states of an aggregated Markov model, subvectors that represent the distributions over the states of the same mode *M*^*i*^ can be naturally described by partitions.

**Definition 2.3 (Partitioned vectors, multi-indices)**

A multi-index is any vector $\alpha =(\alpha_{1},\ldots ,\alpha_{d})\in N^{d}$. We define the absolute value $|\alpha |=\sum_{i=1}^{d}\alpha_{i}$ and denote dim⁡(α)=d the dimension of ***α***.

A vector *v* is partitioned by a multi-index ***α*** if

$$v_{\alpha }:=(v^{1};\ldots ;v^{i};\ldots ;v^{\dim(\alpha )})$$

and for each *i* we have $v^{i}\in R^{\alpha_{i}}$. Selection of the *i*th partition of *v*_***α***_ is written as

$$v_{\alpha }(i)=v^{i}.$$

The vector space of ***α***-partitioned vectors *v*_***α***_ is denoted $R^{\alpha }$.

How distributions *p* over the states of a hierarchical Markov model relate to distributions over the states of $\tilde{M}$ and *Q*^*i*^ can be clarified by the tensor product of partitioned vector spaces.

**Definition 2.4 (Tensor product $R^{m}{⊗}_{m,n}R^{n}$ of *d*-partitioned vector spaces)**

Let $m,n\in N^{d}$, $v_{m}\in R^{m}$, $w_{n}\in R^{n}$ be *d*-partitioned vectors. Then the tensor product *u*_***m***⋅***n***_ of *d*-partitioned vectors *v*_***m***_ and *w*_***n***_ is defined by

2.3 $$u_{m\cdot n}:=v_{m}{⊗}_{m,n}w_{n}:=(v^{1}⊗w^{1};\ldots ;v^{i}⊗w^{i};\ldots ;v^{d}⊗w^{d}),$$

with the component-wise product ***m***⋅***n*** of ***m*** and ***n***. With the tensor product ‘⊗_***m***,***n***_’ we obtain the vector space

$$R^{m}{⊗}_{m,n}R^{n}$$

of the *d*-partitioned vector spaces $R^{m}$ and $R^{n}$.

**Remark 2.1**

We make some remarks regarding the interpretation of definition 2.4:

— It can be easily verified that ‘⊗_***m***,***n***_’ fulfils the properties of a tensor product on the vector space $R^{m}{⊗}_{m,n}R^{n}$.
— Vectors $u_{m\cdot n}\in R^{m}{⊗}_{m,n}R^{n}$ can be written as linear combinations
  2.4 $$u_{m\cdot n}=\sum_{k=1}^{d}\sum_{i=1}^{m_{k}}\sum_{j=1}^{n_{k}}a_{ij}^{k}(v_{m}^{k,i}{⊗}_{m,n}w_{n}^{k,j}),\;a_{i,j}^{k}\in R,$$
  where d=dimm=dimn. By choosing bases {*v*^*k*,*i*^}, *i*=1,…,*m*_*k*_, {*w*^*k*,*j*^}, *j*=1,…,*n*_*k*_, we obtain systems of linearly independent vectors
  $$v_{m}^{k,i}=(0;\ldots ;v^{k,i};\ldots ;0)\in R^{m}$$
  and
  $$w_{n}^{k,j}=(0;\ldots ;w^{k,j};\ldots ;0)\in R^{n}.$$
  Thus, from (2.4) it is easy to see that
  $$R^{m}{⊗}_{m,n}R^{n}≅R^{m\cdot n},$$
  where ***m***⋅***n*** again denotes the component-wise product of ***m*** and ***n***.

### (b) A hierarchical Markov model for modal gating

Based on the block structure (1.1) of $\tilde{M}$, we now show how a transition matrix for the full model can be calculated from its components $\left(({\tilde{m}}_{0},\tilde{M}),{\left(p^{i},Q^{i}\right)}_{i=1}^{n_{M}}\right)$. Let ***m*** and ***n*** be the multi-indices defined above. The transitions within the modes *M*^*i*^ are represented in the full model by block matrices $M^{i,i}={\tilde{M}}^{i,i}⊕Q^{i}\in R^{m_{i}n_{i}\times m_{i}n_{i}}$. It follows that $\dim M^{i,i}=m_{i}n_{i}$. Moreover, we define the matrix of initial conditions for a transition from *Q*^*i*^ to *Q*^*j*^ by

2.5 $$P^{i,j}=u_{n_{i}}^{T}⊗p^{j}=p^{j}⊗u_{n_{i}}^{T},$$

where the row vector $p^{j}\in R^{1\times n_{j}}$ is the initial condition for *Q*^*j*^ from definition 2.5, and $u_{n_{i}}^{T}\in R^{n_{i}\times 1}$ is a column vector of ones. We observe that $P^{i,j}\in R^{n_{i}\times n_{j}}$ so that, for *i*≠*j* we have $M^{i,j}={\tilde{M}}^{i,j}⊗P^{i,j}\in R^{m_{i}n_{i}\times m_{j}n_{j}}$. We can now define the components of a continuous-time hierarchical Markov model and calculate its infinitesimal generator:

**Definition 2.5 (Components of a continuous-time hierarchical Markov model)**

A continuous-time hierarchical Markov model (with a two-level hierarchy) is specified by the components $\left(({\tilde{m}}_{0},\tilde{M}),{\left(p^{i},Q^{i}\right)}_{i=1}^{n_{M}}\right)$:

— An infinitesimal generator $\tilde{M}$ of a Markov model with initial distribution ${\tilde{m}}_{0}$ with aggregates of states *M*^*i*^, *i*=1,…,*n*_*M*_. The *M*^*i*^ are referred to as modes.
— For each mode, a Markov model with infinitesimal generator *Q*^*i*^ and initial distribution *p*^*i*^.

Then the infinitesimal generator *M* of the aggregated model for modal gating is calculated as follows:

2.6 M=(M~1,1⊕Q1|M~1,2⊗P1,2|…|M~1,nM⊗P1,nMM~2,1AA⊗P2,1|M~2,2⊕Q2|…|M~2,nM⊗P2,nM⋮⋱⋮⋮⋱⋮⋮⋱⋮M~nM,1⊗PnM,1|………|M~nM,nM⊕QnM).

It is straightforward to generalize this definition recursively to an arbitrary number of hierarchies. From definition 2.4 and (2.3), we know that an arbitrary distribution *p* over the states of the full model can be represented by a linear combination of tensor products of the form (2.3). We now require for initial distributions that they should arise from a single tensor product of initial distributions over the states of $\tilde{M}$ and initial distributions over the states of the *Q*^*i*^.

**Definition 2.6 (Initial distribution over the states of a hierarchical Markov model)**

Let *v*_***m***_ be the initial distribution over the states of the top-level model $\tilde{M}$ and *w*_***n***_, a vector whose components *w*^*i*^ are initial distributions over the states of the models *Q*^*i*^. Then the initial distribution $p_{m\cdot n}^{0}$ over the states of the full model *M* is calculated by the tensor product ‘⊗_***m***,***n***_’ introduced in definition 2.4:

2.7 $$p_{m\cdot n}^{0}=v_{m}{⊗}_{m,n}w_{n}=(v^{1}⊗w^{1};\ldots ;v^{i}⊗w^{i};\ldots ;v^{n_{M}}⊗w^{n_{M}}).$$

**Remark 2.2**

We make some remarks regarding the interpretation of definition 2.6:

— Note that whereas *v*_***m***_ is a stochastic vector, *w*_***n***_ is not. It is easy to see that $p_{m\cdot n}^{0}$ is a stochastic vector.
— Algebraically, definition 2.6 constrains initial distributions to so-called pure tensors which can be written as a single tensor product rather than a linear combination of tensor products.
— Statistically, definition 2.6 says that for the initial distribution the probabilities of being in a state ${\tilde{M}}_{j}^{i}$ and a state $Q_{k}^{i}$ are stochastically independent: the joint probability of being in ${\tilde{M}}_{j}^{i}$ and $Q_{k}^{i}$ is the product of the individual probabilities (2.7).

It is an interesting question if the time-dependent solution *p*_***m***⋅***n***_(*t*) or the stationary distribution of the full model *M* remain in the form *p*_***m***⋅***n***_(*t*)=*v*_***m***_(*t*)⊗_***m***,***n***_*w*_***n***_(*t*) for *t*>0. In fact, this is generally not the case.

**Remark 2.3 (Caution)**

In most situations, *p*_***m***⋅***n***_(*t*) cannot be written as a pure tensor *p*_***m***⋅***n***_(*t*)=*v*_***m***_(*t*)⊗_***m***,***n***_*w*_***n***_(*t*) for *t*>0. As discussed in proposition 4.4, we obtain a solution (*v*_***m***_(*t*)⊗_***m***,***n***_*π*_***n***_) for a solution *v*_***m***_(*t*) of $\tilde{M}$ and a vector *π*_***n***_ of stationary solutions *π*^*i*^ of *Q*^*i*^ if and only if we choose initial conditions *p*^*i*^=*π*^*i*^ for all *Q*^*i*^.

### (c) Example

As an example for the construction of the infinitesimal generator *M* from the components $(({\tilde{m}}_{0},\tilde{M}),{\left(p^{i},Q^{i}\right)}_{i=1}^{n_{M}}),$ we present a model that will be used in §3 for experimental data from the inositol trisphosphate receptor (IP_3_R).

Let the infinitesimal generator for the switching between modes be
2.8
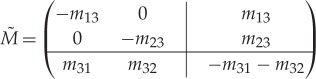

and the models representing the intra-modal kinetics

2.9 $$Q^{1}=\left(\begin{array}{cc} -q_{12}^{1} & q_{12}^{1}\\ q_{21}^{1} & -q_{21}^{1} \end{array}\right)\;\text{and}\;Q^{2}=\left(\begin{array}{cccc} -q_{12}^{2} & q_{12}^{2} & 0 & 0\\ q_{21}^{2} & -q_{21}^{2}-q_{23}^{2}-q_{24}^{2} & q_{23}^{2} & q_{24}^{2}\\ 0 & q_{32}^{2} & -q_{32}^{2} & 0\\ 0 & q_{42}^{2} & 0 & -q_{42}^{2} \end{array}\right)$$

with initial conditions

2.10 $$p^{1}=(p_{1}^{1},p_{2}^{1})\;\text{and}\;p^{2}=(p_{1}^{2},p_{2}^{2},p_{3}^{2},p_{4}^{2}).$$

Then
2.11
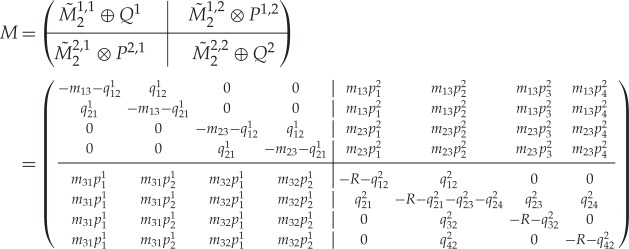

with *R*:=*m*_31_+*m*_32_.

### (d) Parametrizing the model with experimental data

In order to parametrize the components $\left(({\tilde{m}}_{0},\tilde{M}),{\left(p^{i},Q^{i}\right)}_{i=1}^{n_{M}}\right)$ of our model, the infinitesimal generators $\tilde{M}$ and *Q*^*i*^ have to be inferred from ion channel data. We assume that the original data, a sequence of current measurements recorded with a constant sampling interval *τ*, have been statistically analysed so that they have the form of figure 1. Apart from visual inspection, mode changes have been investigated based on calculating the open probability within a window of a certain number of data points. One problem with these methods based on moving averages is that—depending on the window size—instantaneous jumps are transformed to gradual transitions so that the transitions between modes cannot be localized very accurately. By contrast, the heuristic method by Ionescu *et al.* [9] localizes switching events at specific data points but the uncertainty of the segmentation into different modes cannot be quantified. By contrast, the method by Siekmann *et al.* [6] calculates probability distributions for the position of each transition between different modes so that for each detected transition between different modes comprehensive information on the uncertainty is available. After a time series has been segmented each measurement is classified as open (O) or closed (C) and it has also been determined in which mode *M*^*i*^ the channel was at this point in time. From the results of a probabilistic method such as Siekmann *et al.* [6] rather than assigning a particular mode to each data point, it is possible to calculate a probability distribution for the different modes. This may improve the results for datasets where mode changes cannot be localized very accurately. The Markov model $\tilde{M}$ is then inferred from the sequence *S*^*k*^ of modes *M*^*i*^, whereas the models *Q*^*i*^ are parametrized from sequences of *T*^*k*^ that are representative of a particular mode. For example, in figure 1, the five data points between *j*_*n*_ and *j*_*n*+1_ could be used for inferring the model *Q*^2^ representing the stochastic opening and closing within mode M^2^.

All models are parametrized with the Bayesian method developed in Siekmann *et al.* [19,20] or, alternatively, any other algorithm for fitting Markov models to single channel data. For inferring the infinitesimal generator $\tilde{M}$ the likelihood has the form

2.12 $$P((S^{k})|\tilde{M})=\tilde{\mu }\cdot P_{S^{1}}\cdot \exp(\tilde{M}\tau )\cdot P_{S^{2}}\cdot \cdots \cdot \exp(\tilde{M}\tau )\cdot P_{S^{N}}\cdot u^{T},$$

where (*S*^*k*^) is a sequence of observations of modes *M*^*i*^ separated by the sampling interval *τ*, $\tilde{M}$ is the infinitesimal generator of an aggregated Markov model, $\tilde{\mu }$ is the stationary distribution of $\tilde{M}$ and *u*^*T*^ is a column vector of ones. The matrices *P*_*S*^*k*^_ project to the states of the model that represent the mode observed at data point *k*. For example,
2.13
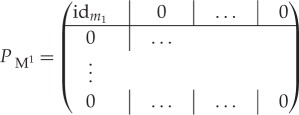

with the same block structure as in (1.1) projects to states representing mode M^1^, the other projection matrices *P*_*S*^*i*^_ are defined equivalently. The likelihood for inferring the infinitesimal generators *Q*^*i*^ from representative segments of *T*^*k*^ of open (O) and closed (C) events (figure 1) is analogous to (2.12). Missed events, see Hawkes and co-workers [21–23] and the references therein, are not considered because they are not relevant for this approach. The method is discussed in detail in Siekmann *et al.* [19,20].

## 3. Data-driven modelling of modal gating

Our new framework enables us to easily construct and parametrize models for modal gating following a transparent iterative process:

(i) Infer the stochastic process *S*^*k*^ of switching between modes *M*^*i*^ (figure 1).
(ii) Model the process *S*^*k*^ of mode switching by parametrizing an infinitesimal generator $\tilde{M}$ (figure 3*a*).
(iii) From segments of *T*^*k*^ representative for the opening of closing within each of the modes M^1^, M^2^, … (figure 3*b*) parametrize infinitesimal generators *Q*^1^, *Q*^2^, …
(iv) Choose initial distributions ${\tilde{m}}_{0}$ and *p*^*i*^ and combine all components $\left(({\tilde{m}}_{0},\tilde{M}),{\left(p^{i},Q^{i}\right)}_{i=1}^{n_{M}}\right)$ by calculating the infinitesimal generator *M* of the full model (figure 4).

Inferring $\tilde{M}$ and *Q*^*i*^ using the Bayesian approach briefly described in §2d ensures that the resulting model will be highly parsimonious because at each step a model with the optimal number of parameters for representing stochastic switching between modes, and opening and closing within modes, is determined. We demonstrate the practical implementation of this process using data collected by Wagner & Yule [15] and compare the results with our previously published model of the same dataset [12].

### (a) Step (i): statistical analysis of modal gating

**Figure 5.**
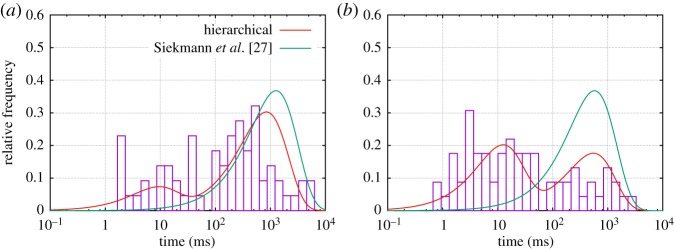
Empirical sojourn time distributions for both modes M^1^ and M^2^ for type II IP_3_R for for 10 μM IP_3_, 5 mM ATP and 0.05 μM Ca^2+^. Whereas the hierarchical model can resolve (by using a four-state model) the widespread distributions of both M^1^ and M^2^, the model from Siekmann *et al.* [12] can only capture one characteristic sojourn time due to the fact that only one pair of transition rates has been used to connect the submodels for mode M^1^ and M^2^. Sojourn time distribution in (*a*) M^1^ and (*b*) M^2^.

Previously, we have statistically analysed mode switching exhibited in the data by Wagner & Yule [15] and found two modes, the nearly inactive mode M^1^ with a very low open probability and the highly active mode M^2^ with *P*_*O*_≈70% (see Siekmann *et al.* [6] for details). As illustrated in figure 1, we have a stochastic sequence of events M^1^ and M^2^ that are separated by a sampling interval *τ*=0.05 ms. We have results from two types of the inositol trisphosphate receptor (type I IP_3_R and type II IP_3_R) for various calcium concentrations (Ca^2+^), 0.01 μM, 0.05 μM and 5 μM, at fixed concentrations of 10 μM inositol trisphosphate (IP_3_) and 5 mM adenosine trisphosphate (ATP). Empirical histograms of the sojourn times in M^1^ and M^2^ for all except one dataset indicate that whereas time spent in the active mode M^2^ may be represented satisfactorily by one state, accurately representing sojourn times in the nearly inactive mode M^1^ seems to require at least two states (e.g. figure 2). Whereas one state accounts for the support of the sojourn time density in mode M^2^ (figure 2*b*), the more widespread sojourn time density in mode M^1^ is better approximated by two states (figure 2*a*). Thus, for five of our six datasets we parametrize $\tilde{M}$ with the structure of (2.8). For one dataset (type II IP_3_R at 0.05 μM Ca^2+^), the histograms suggest that we need a model with two states representing M^1^ and two states representing M^2^ (figure 5). Thus, for these data we use the following infinitesimal generator:
3.1
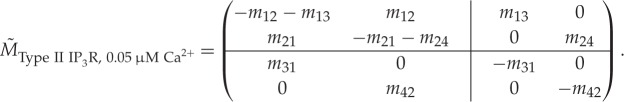

It may seem that the mode switching dynamics of type II IP_3_R is represented here with two different model structures. But, in fact, we can obtain the model structure from (3.1) by simply adding an additional M^2^ state to the models for 0.01 μM Ca^2+^ and 5 μM Ca^2+^ such that transition rates entering this state vanish. The interpretation of this is that the additional state representing long sojourns in M^2^ observed for 0.05 μM Ca^2+^ —although present in the model—is never visited at the other ligand concentrations.

### (b) Step (ii): parametrizing $\tilde{M}$

Fitting $\tilde{M}$ to a time series *S*^*k*^ of M^1^ and M^2^ using our MCMC method [19,20] is a challenging problem. Because in a time series of a few hundred thousand up to about a million data points, the number of transitions between the two modes is only in the order of hundreds, the data from which the rate constants have to be inferred are effectively very limited—despite the large number of data points. An example of a convergence plot shown in electronic supplementary material, figure S1, demonstrates that values of the two rates, *m*_13_ and *m*_23_, alternate. This is due to symmetry in the model structure chosen for the model $\tilde{M}$ where the two states $M_{1}^{1}$ and $M_{2}^{1}$ can be swapped without changing the model. This effect can be removed by considering only one mode of the multi-modal posterior, in this case by considering only samples where *m*_31_ exceeds a certain threshold. Nevertheless, even after this correction some parameters such as the rate *m*_23_ show a high degree of uncertainty indicated by a widespread marginal distribution (electronic supplementary material, figure S1). Mean values and standard deviations of the distributions of the model parameters are summarized in electronic supplementary material, tables S1 and S2.

### (c) Step (iii): parametrizing *Q*^1^ and *Q*^2^

In our previous study [12], we have already fitted a model with two states to representative segments of the inactive mode M^1^ and a model with four states for representing M^2^, see (2.9) for the form of the infinitesimal generators *Q*^1^ and *Q*^2^. Interestingly, we could show that *Q*^1^ and *Q*^2^ were independent of the concentrations of IP_3_, ATP and Ca^2+^. The parameter values from the Supplementary Material of Siekmann *et al.* [12] are reproduced here for convenience (electronic supplementary material, table S3).

### (d) Step (iv): the generator *M* of the full model

After the models $\tilde{M}$, *Q*^1^ and *Q*^2^ have been obtained, we finally need to specify the initial distributions ${\tilde{m}}_{0}$, *p*^1^ and *p*^2^. Consistent with the experimental assumption that recording of the data was started when the channel had reached steady state, we set ${\tilde{m}}_{0}=\tilde{\mu }$, *p*^1^=*π*^1^ and *p*^2^=*π*^2^, where $\tilde{\mu }$, *π*^1^ and *π*^2^ are the stationary distributions of $\tilde{M}$, *Q*^1^ and *Q*^2^, respectively. After all components $\left(({\tilde{m}}_{0},\tilde{M}),{\left(p^{i},Q^{i}\right)}_{i=1}^{n_{M}}\right)$ of our model have been specified, the infinitesimal generator *M* of the full model can be calculated using (2.6).

### (e) Results

Owing to the problems with fitting the infinitesimal generator $\tilde{M}$ (2.8) mentioned in §3b, one may ask if a simpler two-state model representing the dynamics of modal gating would be preferable. However, the ability of a three-state model to approximate the sojourn distribution of the nearly inactive mode M^1^ more accurately (figure 2*a*) was found to be crucial for obtaining a better fit of the closed time distribution in comparison with the model from Siekmann *et al.* [12] (figure 2*c*). That the model structure of the hierarchical model proposed here is better able to capture the properties of the entire time series data seems even more convincing because it has—unlike the original model from Siekmann *et al.* [12]—been built without directly fitting to the time series at any step of its construction.

In electronic supplementary material, figure S2, we show that the bimodal closed time distribution observed for some combinations of ligand concentrations arises due to the mixing of the closed time distributions within nearly inactive mode M^1^ and active mode M^2^ both of which only have one distinct maximum.

Stronger differences between both models are observed for a dataset collected from type II IP_3_R for 10 μM IP_3_, 5 mM ATP and 0.05 μM Ca^2+^. For this experimental condition, the effect of modal gating can be observed without statistical analysis (electronic supplementary material, figure S3a). Figure 5 shows that both modes M^1^ and M^2^ exhibit a widespread distribution of sojourn times which can only approximately be captured by a four-state model with two states each for both M^1^ and M^2^. Whereas the new hierarchical model can approximate the empirical distributions of both modes relatively well, the model from Siekmann *et al.* [12] fails due to the fact that only one characteristic sojourn time for each mode can be captured by the pair of transition rates accounting for modal gating in this model (figure 5).

**Figure 6.**
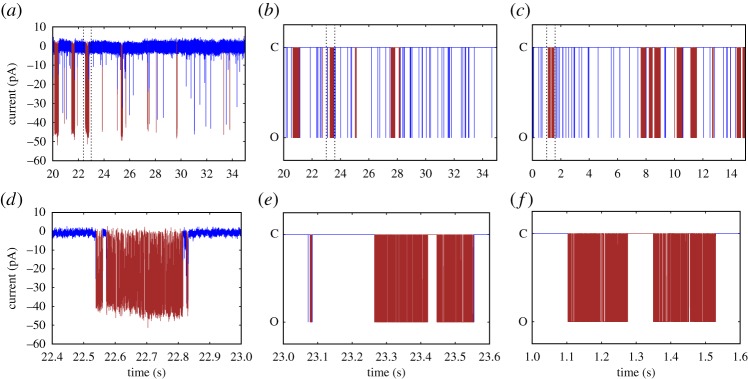
Comparison of a segment of data from type II IP_3_R recorded at recorded at 10 μM IP_3_, 5 mM ATP and 0.05 μM (*a*,*d*) with simulations of the hierarchical model presented here and the model from Siekmann *et al.* [12]. The colour of the line indicates if the channel is in the nearly inactive mode M^1^ or the active mode M^2^. As expected from the dwell time distributions of the two modes (figure 2), the model from Siekmann *et al.* [12] shows too many long sojourns in the active mode M^2^ as well as in the inactive mode M^1^ ((*c*) and (*f*)). By contrast, both long as well as short visits to both modes are seen in the sample path generated for the hierarchical model which is closer to what is observed in the data ((*b*) and (*e*)). (*a*) Data, (*b*) hierarchical model (HM), (*c*) Siekmann *et al.* [12] (SM), (*d*) data (detail), (*e*) HM (detail) and (*f*) SM (detail).

Owing to the failure to account for the modal sojourn time distributions, we expect the model from Siekmann *et al.* [12] to reproduce the kinetics observed in the data much less accurately than the new hierarchical model. In order to illustrate this, we simulated both the Siekmann *et al.* [12] model and the new model and compared them with a segment of experimental data of the same length (figure 6). The sample path was plotted in blue when the channel was in mode M^1^, whereas it was plotted in brown when the channel was in mode M^2^. The same colours were used for colouring the data based on the results of the statistical analysis from Siekmann *et al.* [6]. In the data segment shown here, both dwell times in the active mode M^2^ of about 0.2–0.5 s are observed as well as very brief sojourns of a few milliseconds. Consistent with the dwell time distribution (figure 2), the long but not the short sojourns in the active mode M^2^ are captured by the model from Siekmann *et al.* [12], whereas the hierarchical model developed in this study reproduces both long and short sojourns in this mode. Interestingly, as we show in the electronic supplementary material, for this particular dataset the channel seems to change its behaviour at an even slower time scale by spontaneously increasing the observed prevalence in M^2^ for an extended period of time before returning to the initial level of activity (electronic supplementary material, figure S3a).

## 4. Mathematical analysis of the hierarchical Markov model

In the previous section, we demonstrated that the hierarchical Markov model introduced in §2 provides a statistically efficient framework for systematically building models for modal gating. Now, we focus on some interesting aspects of the mathematical structure of the hierarchical Markov model and show that many important properties of the infinitesimal generator *M* of the full model can be derived from the components $\left(({\tilde{m}}_{0},\tilde{M}),{\left(p^{i},Q^{i}\right)}_{i=1}^{n_{M}}\right)$ of the model.

In §4a, we calculate the eigenvalues of *M*. The spectrum of *M* consists of two parts: the eigenvalues of $\tilde{M}$ and a subset of the eigenvalues of the blocks $M^{i,i}={\tilde{M}}^{i,i}⊕Q^{i}$. But whereas the eigenvalues of the submatrices ${\tilde{M}}^{i,i}$ appear in the spectrum of the submatrices *M*^*i*,*i*^, they are not eigenvalues of the full model *M*.

From a modelling point of view, it is an important question if properties of the components $\left(({\tilde{m}}_{0},\tilde{M}),{\left(p^{i},Q^{i}\right)}_{i=1}^{n_{M}}\right)$ are preserved when they are combined in the full model. In §4b, we demonstrate that the sojourn time distribution in the states representing a particular mode in the model $\tilde{M}$ is preserved for the analogous distribution calculated for the augmented state space of *M*.

When the initial distributions *p*^*i*^ coincide with the stationary distributions, *p*^*i*^=*π*^*i*^, we calculate the full time-dependent solution and the stationary distribution of *M* from the components $\left(({\tilde{m}}_{0},\tilde{M}),{\left(p^{i},Q^{i}\right)}_{i=1}^{n_{M}}\right)$ of the hierarchical Markov model (§4c).

### (a) Eigenvalues

Before we calculate the eigenvalues for general infinitesimal generators *M* of the full model, we remark that in most cases relevant for ion channel modelling we may assume that the matrices $\tilde{M}$ and *Q*^*i*^ appearing in our model are diagonalizable—this is implied by the so-called detailed balance conditions:

4.1 $$\pi^{i}q_{ij}=\pi^{j}q_{ji},$$

where *π* is the stationary distribution of an infinitesimal generator *Q*=(*q*_*ij*_). A matrix *Q*=(*q*_*ij*_) with (4.1) is diagonalizable with real eigenvalues because by choosing the transformation matrix *diag*(*π*)^1/2^ it is similar to a symmetric real matrix. Detailed balance is usually assumed to hold for ion channel models because it can be related to thermodynamic reversibility of the transitions between different states in the model. Note that (4.1) holds automatically if the adjacency graph of the states of a Markov model is acyclic. This follows from Kolmogorov's criterion [24], see theorem 1.8 of [25] for a more recent statement of the continuous-time version. Thus, in particular, all infinitesimal generators $\tilde{M}$ and *Q*^*i*^ considered in this article satisfy detailed balance.

**Proposition 4.1 (Eigenvalues and eigenvectors of *M* assuming detailed balance)**

*We assume that the matrices* $\tilde{M}$ *and* *Q*^*i*^ *of a hierarchical Markov model fulfil the detailed balance conditions* (4.1).

(i) *Let* *ζ* *be an eigenvalue of the matrix* $\tilde{M}$ *and* *v*^*T*^_***m***_ *a right eigenvector associated with* *ζ*. *Then* *ζ* *is also an eigenvalue of the full model* *M* *with associated right eigenvector* *v*^*T*^_***m***_⊗*u*^*T*^_***n***_, *where* *u*^*T*^_***n***_ *is a vector of* |***n***| *ones*.
(ii) *Moreover, all* $\nu =\tilde{\zeta }+\lambda ,$ *where* $\tilde{\zeta }$ *is an eigenvalue of* ${\tilde{M}}^{i,i}$ *and* *λ*≠0 *is an eigenvalue of* *Q*^*i*^, *are eigenvalues of the full model* *M*. *If* ${\tilde{w}}^{i}$ *is a left eigenvector of the submatrix* *M*^*i*,*i*^ *associated with the eigenvalue* *ν*, $w_{m}=(0;\ldots ;0;{\tilde{w}}^{i};0;\ldots ;0)$ *with* $w(i)={\tilde{w}}^{i}$ *and* *w*(*j*)=0, *i*≠*j* *is a left eigenvector of* *M* *associated with* *ν*.

**Proof.**

Detailed balance implies that $\tilde{M}$ and the *Q*^*i*^ are diagonalizable with real eigenvalues. In particular, all matrices have full sets of eigenvectors. This enables us to construct eigenvectors of the infinitesimal generator *M* of the full model from the eigenvectors of $\tilde{M}$ and the *Q*^*i*^.

(i) We need to show that *M*(*v*^*T*^_***m***_⊗_***m***,***n***_*u*^*T*^_***n***_)=*ζ*(*v*^*T*^_***m***_⊗_***m***,***n***_*u*^*T*^_***n***_). Let [*M*(*v*^*T*^_***m***_⊗_***m***,***n***_*u*^*T*^_***n***_)]^*i*^ denote the *i*th component of the partitioned vector. Here, *v*^*T*^_***m***_⊗_***m***,***n***_*u*^*T*^_***n***_ is a tensor product that is consistent with the partitions ***m*** and ***n*** as in (2.3) (definition 2.3). We calculate
  $${\left[M(v_{m}^{T}{⊗}_{m,n}u_{n}^{T})\right]}^{i}=({\tilde{M}}^{i,i}⊕Q^{i})({\left(v^{i}\right)}^{T}⊗u_{n_{i}}^{T})+\sum_{k\neq i}({\tilde{M}}^{i,k}⊗P^{i,k})({\left(v^{k}\right)}^{T}⊗u_{n_{k}}^{T}).$$
  Using the compatibility condition of matrix multiplication and tensor product (A.2) we calculate
  $${\left[M(v_{m}^{T}{⊗}_{m,n}u_{n}^{T})\right]}^{i}=({\tilde{M}}^{i,i}{\left(v^{i}\right)}^{T}⊗u_{n_{i}}^{T}+{\left(v^{i}\right)}^{T}⊗Q^{i}u_{n_{i}}^{T})+\sum_{k\neq i}({\tilde{M}}^{i,k}{\left(v^{k}\right)}^{T}⊗P^{i,k}u_{n_{k}}^{T}).$$
  Noting that $Q^{i}u_{n_{i}}^{T}=0$ and $P^{i,k}u_{n_{k}}^{T}=u_{n_{i}}^{T}$, we finally get
  $${\left[M(v_{m}^{T}{⊗}_{m,n}u_{n}^{T})\right]}^{i}=\sum_{k=1}^{n_{M}}{\tilde{M}}^{i,k}{\left(v^{k}\right)}^{T}⊗u_{n_{i}}^{T}=\zeta ({\left(v^{i}\right)}^{T}⊗u_{n_{i}}^{T}).$$
  Because this holds for all blocks we obtain the desired result.
(ii) All except for the *i*th block of *w* are zero, so we get
  $$wM=({\tilde{w}}^{i}[{\tilde{M}}^{i,1}⊗P^{1,i}];\ldots ;{\tilde{w}}^{i}[{\tilde{M}}^{i,i}⊕Q^{i}];\ldots ;{\tilde{w}}^{i}[{\tilde{M}}^{i,n_{M}}⊗P^{n_{M},i}]).$$
  Because ${\tilde{w}}^{i}$ is an eigenvector of ${\tilde{M}}^{i,i}⊕Q^{i}$ we know that ${\tilde{w}}^{i}({\tilde{M}}^{i,i}⊕Q^{i})=\nu {\tilde{w}}^{i}$. For *w* to be an eigenvector, it remains to be shown that all other blocks vanish. Let *u* be a left eigenvector of ${\tilde{M}}^{i,i}$ associated with the eigenvalue $\tilde{\zeta }$ and *v* a left eigenvector of *Q*^*i*^ associated with the eigenvalue *λ*. Then ${\tilde{w}}^{i}$ can be written as ${\tilde{w}}^{i}=u⊗v$ according to (A.3). Substituting this and $P^{i,k}=p^{k}⊗u_{n_{i}}^{T}$, *k*≠*i*, we calculate
  4.2 $$(u⊗v)[{\tilde{M}}^{1,k}⊗p^{k}⊗u_{n_{i}}^{T}]=u({\tilde{M}}^{1,k}⊗p^{k})⊗vu_{n_{i}}^{T}.$$
  The term $vu_{n_{i}}^{T}$ is the standard scalar product $\left\langle v^{T},u_{n_{i}}^{T}\right\rangle$ of the vectors *v*^*T*^ and $u_{n_{i}}^{T}$. Because the row sums of *Q*^*i*^ are zero, $u_{n_{i}}^{T}$ is in the right nullspace of *Q*^*i*^. By assumption, *v* is an eigenvector associated with any eigenvalue *λ*≠0. This means that *v* is not in the left nullspace of *Q*^*i*^, so it must be orthogonal to any vector in the right nullspace. It follows that (4.2) vanishes as required.

 ▪

For the general case where the infinitesimal generators of the model $\tilde{M}$ and the submatrices *M*^*i*,*i*^ may not necessarily be diagonalizable we need the Schur decomposition (proposition A.2). The Schur decomposition ensures that the matrix *M* can be transformed to an upper-triangular matrix by a unitary matrix. In the following, we construct a unitary matrix *S* from the components $\left(({\tilde{m}}_{0},\tilde{M}),{\left(p^{i},Q^{i}\right)}_{i=1}^{n_{M}}\right)$ of our model.

**Lemma 4.1 (Unitary matrix *S*)**

*For the components* $\left(({\tilde{m}}_{0},\tilde{M}),{\left(p^{i},Q^{i}\right)}_{i=1}^{n_{M}}\right)$ *of a hierarchical Markov model, let
*

$$T_{\tilde{M}}=\Theta^{*}\tilde{M}\Theta ,\;T_{{\tilde{M}}^{i,i}⊕Q^{i}}={\left(V_{i}⊗W_{i}\right)}^{*}{\tilde{M}}^{i,i}⊕Q^{i}(V_{i}⊗W_{i}),$$

*be the Schur decompositions of* $\tilde{M}$ *and* ${\tilde{M}}^{i,i}⊕Q^{i}$*. Let* ${\bar{u}}_{n_{i}}^{T}=1/\sqrt{n_{i}}u_{n_{i}}^{T}$ *be the vectors obtained by normalizing the vectors of ones* $u_{n_{i}}^{T}$.

(i) *The matrices W*_*i*_ *may be chosen so that they have the form* $W_{i}=({\bar{u}}_{n_{i}}^{T}|{\tilde{W}}_{i})$ *with* ${\tilde{W}}_{i}\in C^{n_{i}\times (n_{i}-1)}$.
(ii) *Let*
  $$\Theta =\left(\begin{array}{c} \Theta^{1}\\ \vdots \\ \Theta^{n_{M}} \end{array}\right)$$
  *be row-partitioned according to the block structure of* $\tilde{M}$ *from (1.1). Then the matrix
*4.3
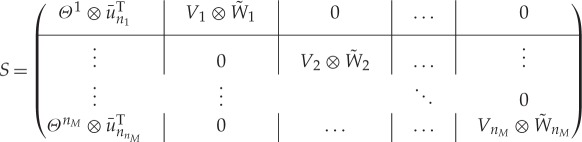

*is unitary.*

**Proof.**

(i) Because the row sums of *Q*^*i*^ vanish, the vector ${\bar{u}}_{n_{i}}^{T}$ is a right eigenvector of *Q*^*i*^ associated with the eigenvalue zero. Without loss of generality, we can choose ${\bar{u}}_{n_{i}}^{T}$ as the first column of *W*_*i*_.
(ii) By construction, all column vectors of *S* are normalized. Thus, it remains to show that they are also pairwise orthogonal. By definition, any two distinct column vectors appearing in the same block of *S* are orthogonal. It is trivial that column vectors from different blocks are orthogonal unless one of the two appears in the first block of *S*. Thus, let *θ*^*T*^ be a column vector of *Θ* and $v_{i}^{T}⊗{\tilde{w}}_{i}^{T}$ be a column vector of any *V*_*i*_⊗*W*_*i*_. With the shorthand for tensor products consistent with partitions (2.3) introduced in definition 2.3, the scalar product 〈⋅,⋅〉 of the two columns is
  $$\left\langle \theta_{m}^{T}{⊗}_{m,n}{\bar{u}}_{n}^{T},{\left(0;\ldots ;v_{i}⊗{\tilde{w}}_{i};\ldots 0\right)}^{T}\rangle =\langle \theta_{m}^{T}(i)⊗{\bar{u}}_{n_{i}}^{T},v_{i}^{T}⊗{\tilde{w}}_{i}^{T}\right\rangle$$
  and due to the zeroes in all except for the *i*th block, all other summands vanish. Noting that 〈*u*,*v*〉=*u*(v*)^*T*^=*u*^*T*^v* can be interpreted as a special case of matrix multiplication (where ‘*’ denotes component-wise complex conjugation) we can use (A.2):
  $$\langle \theta_{m}^{T}(i){⊗}_{m,n}{\bar{u}}_{n_{i}}^{T},v_{i}^{T}⊗{\tilde{w}}_{i}^{T}\rangle =\langle \theta_{m}^{T}(i),v_{i}^{T}\rangle \langle {\bar{u}}_{n_{i}}^{T},{\tilde{w}}_{i}^{T}\rangle .$$
  But because ${\bar{u}}_{n_{i}}^{T}$ appeared as a column in the original unitary matrix *W*_*i*_, the ${\tilde{w}}_{i}^{T}$ are all orthogonal to ${\bar{u}}_{n_{i}}^{T}$ so that the above scalar product vanishes. Thus, the matrix *S* is unitary.

 ▪

**Proposition 4.2 (Eigenvalues of the full model *M*)**

*Let* $\tilde{\zeta }$ *be an eigenvalue of the model* $\tilde{M}$. *Then* $\tilde{\zeta }$ *is also an eigenvalue of the full model* *M*. *Moreover, all* $\nu =\tilde{\zeta }+\lambda ,$ *where* $\tilde{\zeta }$ *is an eigenvalue of* ${\tilde{M}}^{i,i}$ *and* *λ*≠0 *is an eigenvalue of* *Q*^*i*^, *are eigenvalues of the full model* *M*.

**Proof.**

We demonstrate that with the matrix *S* from (4.3), we obtain a Schur decomposition of the matrix *M*. We need to show that *A*=S*MS is upper triangular. The block structure of *S* is rectangular with *n*_*M*_×(*n*_*M*_+1) blocks which means that S* has an (*n*_*M*_+1)×*n*_*M*_ block structure. Thus, the resulting matrix *A* will have (*n*_*M*_+1)×(*n*_*M*_+1) blocks and its diagonal will consist of the eigenvalues of $\tilde{M}$ in the upper left block followed by the remaining eigenvalues from the submatrices ${\tilde{M}}^{i,i}$. We show that all blocks *A*^*i*,*j*^ are upper triangular which implies that *A* is indeed upper triangular. First, a lengthy calculation shows that *A*^1,1^ is a block-wise expanded form of $\Theta^{*}\tilde{M}\Theta$ and thus upper triangular. One can see directly that the remaining elements on the block diagonal are

$$A^{i,i}={\left(V_{i}⊗{\tilde{W}}_{i}\right)}^{*}({\tilde{M}}^{i,i}⊕Q^{i})(V_{i}⊗{\tilde{W}}_{i})$$

and, therefore, all upper triangular.

It remains to show that the lower diagonal blocks *A*^*i*,*j*^ with *i*>*j* vanish. We will demonstrate that the *A*^*i*,*j*^ vanish provided that

4.4 $${\tilde{W}}_{i}^{*}{\bar{u}}_{n_{i}}^{T}=0.$$

Equation (4.4) is just another way of saying that ${\bar{u}}_{n_{i}}^{T}$ is orthogonal to all columns of ${\tilde{W}}_{i}$. But this is true because from Lemma 4.1(i) we know that ${\bar{u}}_{n_{i}}^{T}$ is the first column of *W*_*i*_, so it must be orthogonal to all column vectors of ${\tilde{W}}_{i}$.

We now calculate the subdiagonal blocks *A*^*i*,*j*^, *i*>*j*. First, we calculate the blocks *A*^⋅,1^ on the first block column. We observe that

$${\left(M\cdot S\right)}^{k,1}=({\tilde{M}}^{k,k}⊕Q^{k})(\Theta^{k}⊗{\bar{u}}_{n_{k}}^{T})+\sum_{j\neq k}({\tilde{M}}^{k,j}⊗P^{k,j})(\Theta^{j}⊗{\bar{u}}_{n_{j}}^{T}).$$

Because S* is block diagonal below the first row, we can calculate

$$\begin{array}{rl} A^{k+1,1} & ={\left(S^{*}\cdot M\cdot S\right)}^{k+1,1}={\left(V_{k}⊗{\tilde{W}}_{k}\right)}^{*}({\tilde{M}}^{k,k}⊕Q^{k})(\Theta^{k}⊗{\bar{u}}_{n_{k}}^{T})\\ & \;+\sum_{j\neq k}{\left(V_{k}⊗{\tilde{W}}_{k}\right)}^{*}({\tilde{M}}^{k,j}⊗P^{k,j})(\Theta^{j}⊗{\bar{u}}_{n_{j}}^{T}) \end{array}$$

because in the row (*k*+1)th row of S* for *k*=1,…,*n*_*M*_ only the *k*th block is non-zero. By taking advantage of (A.2), we obtain

$$\begin{array}{rl} A^{k+1,1} & ={\left(V_{k}⊗{\tilde{W}}_{k}\right)}^{*}({\tilde{M}}^{k,k}\Theta^{k}⊗{\bar{u}}_{n_{k}}^{T}+\Theta^{k}⊗Q^{k}{\bar{u}}_{n_{k}}^{T})+\sum_{j\neq k}{\left(V_{k}⊗{\tilde{W}}_{k}\right)}^{*}({\tilde{M}}^{k,j}\Theta^{j}⊗P^{k,j}{\bar{u}}_{n_{j}}^{T})\\ & ={\left(V_{k}⊗{\tilde{W}}_{k}\right)}^{*}({\tilde{M}}^{k,k}\Theta^{k}⊗{\bar{u}}_{n_{k}}^{T})+\sum_{j\neq k}{\left(V_{k}⊗{\tilde{W}}_{k}\right)}^{*}({\tilde{M}}^{k,j}\Theta^{j}⊗{\bar{u}}_{n_{k}}^{T}), \end{array}$$

where we have used $Q^{k}{\bar{u}}_{n_{k}}^{T}=0$ and $P^{k,j}{\bar{u}}_{n_{j}}^{T}={\bar{u}}_{n_{k}}^{T}$. Again using (A.2), we calculate

$$A^{k+1,1}=V_{k}^{*}{\tilde{M}}^{k,k}\Theta^{k}⊗{\tilde{W}}_{k}^{*}{\bar{u}}_{n_{k}}^{T})+\sum_{j\neq k}V_{k}^{*}{\tilde{M}}^{k,j}\Theta^{j}⊗{\tilde{W}}_{k}^{*}{\bar{u}}_{n_{k}}^{T}).$$

This vanishes due to (4.4) as explained above.

For the remaining blocks *A*^*k*+1,*l*+1^, *k*>*l*=1,…,*n*_*M*_−1, we simply calculate

$$\begin{array}{rl} A^{k+1,l+1} & ={\left(V_{k}⊗{\tilde{W}}_{k}\right)}^{*}({\tilde{M}}^{k,l}⊗P^{k,l})(V_{l}⊗{\tilde{W}}_{l})\\ & =(V_{k}^{*}{\tilde{M}}^{k,l}⊗{\tilde{W}}_{k}^{*}P^{k,l})(V_{l}⊗{\tilde{W}}_{l})\\ & =(V_{k}^{*}{\tilde{M}}^{k,l}V_{l})⊗({\tilde{W}}_{k}^{*}P^{k,l}{\tilde{W}}_{l}). \end{array}$$

Replacing *P*^*k*,*l*^ by ${\bar{u}}_{n_{k}}^{T}⊗p^{l}$ (2.5), we get

$$A^{k+1,l+1}=(V_{k}^{*}{\tilde{M}}^{k,l}V_{l})⊗({\tilde{W}}_{k}^{*}{\bar{u}}_{n_{k}}^{T})⊗(p^{l}{\tilde{W}}_{l}),$$

where—due to the term ${\tilde{W}}_{k}^{*}{\bar{u}}_{n_{k}}^{T}$—we again conclude with (4.4) that *A*^*k*+1,*l*+1^ vanishes. ▪

### (b) Sojourn times in modes

We will now investigate the sojourn times within the states that represent the modes *M*^*i*^. The switching between modes is represented by a model with infinitesimal generator $\tilde{M}$ and one can ask if the dynamics is preserved after $\tilde{M}$ is combined with the other components $\left(({\tilde{m}}_{0},\tilde{M}),{\left(p^{i},Q^{i}\right)}_{i=1}^{n_{M}}\right)$ to the generator *M* of the full model . We denote by $f_{{\tilde{M}}^{i}}(t),$ the density function of the sojourn time in mode *M*^*i*^ represented by $\tilde{M}$ and by *f*_*M*^*i*^_(*t*) the sojourn time densities of *M*^*i*^ in the augmented state space of the generator *M* of the full model. If the mode switching dynamics is preserved, the sojourn time densities should be equal and we will show that indeed $f_{M^{i}}(t)=f_{{\tilde{M}}^{i}}(t)$.

**Proposition 4.3 (Modal sojourn times)**

*For* *f*_*M*^*i*^_(*t*), *sojourn time densities within mode* *M*^*i*^ *with an initial distribution* *p*^0^ *as in definition 2.6, we have* $f_{M^{i}}(t)=f_{{\tilde{M}}^{i}}(t)$.

**Proof.**

For simplicity we only treat the case of two aggregates of states, *M*^1^ and *M*^2^. For the sojourn time within *M*^1^ we have

$$f_{M^{1}}(t)=p^{0}M^{2,1}\exp(M^{1,1}t)M^{1,2}u_{m_{2}n_{2}}^{T},$$

where $p^{0}=p_{{\tilde{M}}^{2}}^{0}⊗p_{Q^{2}}^{0}$ is a suitably normalized initial state probability distribution. Substituting from (2.6), we obtain for

$$\begin{array}{rl} \exp(M^{1,1}t)M^{1,2} & =\exp([{\tilde{M}}^{1,1}⊕Q^{1}]t)M^{1,2}\\ & =[\exp({\tilde{M}}^{1,1}t)⊗\exp(Q^{1}t)]({\tilde{M}}^{1,2}⊗P^{1,2}), \end{array}$$

where we have used (A.4) for calculating the matrix exponential. Now,

$$[\exp({\tilde{M}}^{1,1}t)⊗\exp(Q^{1}t)]({\tilde{M}}^{1,2}⊗P^{1,2})=[\exp({\tilde{M}}^{1,1}t){\tilde{M}}^{1,2}]⊗P^{1,2}$$

according to the compatibility of tensor and matrix product (A.2) which will be used repeatedly below. Also note that $\exp(Q^{1}t)\cdot P^{1,2}=P^{1,2}$. Multiplying this on the right by $u_{m_{2}n_{2}}^{T}=u_{m_{2}}^{T}⊗u_{n_{2}}^{T}$ leads to

$$\{[\exp({\tilde{M}}^{1,1}t){\tilde{M}}^{1,2}]⊗P^{1,2}\}(u_{m_{2}}^{T}⊗u_{n_{2}}^{T})=[\exp({\tilde{M}}^{1,1}t){\tilde{M}}^{1,2}u_{m_{2}}^{T}]⊗u_{n_{1}}^{T},$$

where we have evaluated $P^{1,2}u_{n_{2}}^{T}=u_{n_{1}}^{T}$ in the right-most term. Analogous calculations will be carried out automatically below. The above result is now multiplied on the left by $M^{2,1}={\tilde{M}}^{2,1}⊗P^{2,1}$:

$$({\tilde{M}}^{2,1}⊗P^{2,1})[\exp({\tilde{M}}^{1,1}t){\tilde{M}}^{1,2}u_{m_{2}}^{T}]⊗u_{n_{1}}^{T}=[{\tilde{M}}^{2,1}\exp({\tilde{M}}^{1,1}t){\tilde{M}}^{1,2}u_{m_{2}}]⊗u_{n_{2}}^{T}.$$

Finally, we multiply the preceding result on the left by $p^{0}=p_{{\tilde{M}}^{2}}^{0}⊗p_{Q^{2}}^{0}$ and compute

$$\begin{array}{rl} f_{M^{1}}(t) & =(p_{{\tilde{M}}^{2}}^{0}⊗p_{Q^{2}}^{0})[{\tilde{M}}^{2,1}\exp({\tilde{M}}^{1,1}t){\tilde{M}}^{1,2}u_{m_{2}}]⊗u_{n_{2}}^{T}\\ & =[p_{{\tilde{M}}^{2}}^{0}{\tilde{M}}^{2,1}\exp({\tilde{M}}^{1,1}t){\tilde{M}}^{1,2}u_{m_{2}}^{T}]⊗(p_{Q^{2}}^{0}u_{n_{2}}^{T}). \end{array}$$

Now, because $(p_{Q^{2}}^{0}u_{n_{2}}^{T})=1,$ we obtain the desired result:

$$f_{M^{1}}(t)=p_{{\tilde{M}}^{2}}^{0}{\tilde{M}}^{2,1}\exp({\tilde{M}}^{1,1}t){\tilde{M}}^{1,2}u_{m_{2}}^{T}=f_{{\tilde{M}}^{1}}(t).$$

 ▪

### (c) Full solution for *p*^*i*^=*π*^*i*^

If we choose initial conditions *p*^*i*^=*π*^*i*^, where the *π*^*i*^ are stationary distributions of the models *Q*^*i*^, the solution of the full model has a particularly simple form.

**Proposition 4.4 (Full solution for *p*^*i*^= π^*i*^)**

*Let* *v*_***m***_(*t*) *be the time-dependent solution for the initial condition* *w*^0^_***n***_ *and* ${\tilde{\mu }}_{n}$ *be the stationary solution of the infinitesimal generator* $\tilde{M}$ *with their partition* ***m***. *Let* *π*^*i*^, *i*=1,…,*n*_*M*_ *be the stationary distributions of* *Q*^*i*^ *or written as a partitioned vector*, *π*_***n***_ *with its partition* ***n***. *If for each generator* *Q*^*i*^ *we set* *p*^*i*^=*π*^*i*^ *and we choose an initial distribution* $p_{m\;\cdot \;n}^{0}=v_{m}^{0}{⊗}_{m,n}\pi_{n}$ *consistent with definition* 2.6, *the solution* *p*_***m*** ⋅ ***n***_(*t*) *of the full model is*

4.5 $$p_{m\;\cdot \;n}(t)=v_{m}(t){⊗}_{m,n}\pi_{n}=(v^{1}(t)⊗\pi^{1};\ldots ;v^{i}(t)⊗\pi^{i};\ldots ;v^{n_{M}}(t)⊗\pi^{n_{M}}).$$

*By taking the limit* t→∞, *we obtain the stationary distribution*

4.6 $$\mu_{m\;\cdot \;n}={\tilde{\mu }}_{m}{⊗}_{m,n}\pi_{n}=({\tilde{\mu }}^{1}⊗\pi^{1};\ldots ;{\tilde{\mu }}^{i}⊗\pi^{i};\ldots ;{\tilde{\mu }}^{n_{M}}⊗\pi^{n_{M}}).$$

**Remark 4.1**

The stationary distribution (4.6) is independent of the initial distribution $p_{m\cdot n}^{0}$, so, for *p*^*i*^=*π*^*i*^, we converge to the stationary distribution (4.6) also for $p_{m\cdot n}^{0}=(v_{m}^{0}{⊗}_{m,n}w_{n}^{0})$ with *w*^0^_***n***_≠*π*_***n***_ and even for arbitrary initial conditions $p_{m\cdot n}^{0}$ that are inconsistent with definition 2.6.

**Proof.**

That (4.5) is a solution can be shown by substituting *p*_***m***⋅***n***_(*t*)=*v*_***m***_(*t*)⊗_***m***,***n***_*π*_***n***_ into

4.7 $$\frac{dp(t)}{dt}=p(t)M,$$

where *M* is the generator of the full model (2.6). First, we calculate the left-hand side:

4.8 $$\begin{array}{rl} \frac{dp_{m\cdot n}(t)}{dt} & =\frac{d(v_{m}(t){⊗}_{m,n}\pi_{n})}{dt}\\ & =\left(\frac{dv_{m}(t)}{dt}\right){⊗}_{m,n}\pi_{n}\\ & =(v_{m}(t)\tilde{M}){⊗}_{m,n}\pi_{n}, \end{array}$$

where the last equality (4.8) follows because *v*_***m***_(*t*) is a solution of the model generated by $\tilde{M}$.

We now show that we also obtain (4.8) from the right-hand side of (4.7). For the *i*th component [*p*_***m***⋅***n***_(*t*)⋅*M*]^*i*^, we calculate

$${\left[p_{m\cdot n}(t)\cdot M\right]}^{i}=(v^{i}(t)⊗\pi^{i})({\tilde{M}}^{i,i}⊕Q^{i})+\sum_{j\neq i}(v^{j}(t)⊗\pi^{j})({\tilde{M}}^{j,i}⊗P^{j,i}).$$

For the first summand, the contribution of *Q*^*i*^ vanishes because of *π*^*i*^*Q*^*i*^=0

4.9 $$(v^{i}(t)⊗\pi^{i})({\tilde{M}}^{i,i}⊕Q^{i})=(v^{i}(t){\tilde{M}}^{i,i})⊗\pi^{i}+v^{i}(t)⊗\pi^{i}Q^{i}=(v^{i}(t){\tilde{M}}^{i,i})⊗\pi^{i}.$$

Because of *π*^*j*^*P*^*j*,*i*^=*π*^*i*^, the second summand simplifies to

4.10 $$\sum_{j\neq i}(v^{j}(t)⊗\pi^{j})({\tilde{M}}^{j,i}⊗P^{j,i})=\sum_{j\neq i}(v^{j}(t){\tilde{M}}^{j,i})⊗\pi^{i}.$$

With (4.9) and (4.10), we derive for each component:

$${\left[p_{m\;\cdot \;n}(t)\cdot M\right]}^{i}=\sum_{i=1}^{n_{M}}(v^{j}(t){\tilde{M}}^{j,i})⊗\pi^{i}.$$

This means that the right-hand side of (4.7) is indeed of the form (4.8) which confirms that (4.5) is a solution. ▪

## 5. Conclusion

We have proposed a new model for representing modal gating, the spontaneous switching of ion channels between different levels of activity. The model is suitable for modelling channels with an arbitrary number of modes and is capable of representing both the probabilistic opening and closing within modes as well as the stochastic switching between modes that regulates these dynamics.

### (a) Modular representation of modal gating

In comparison with previous studies, the model presented here incorporates modal gating in a much more transparent way. Ullah *et al.* [11] developed their model of the IP_3_R from a binding scheme. First, the authors determined the set of open and closed model states from a statistical model selection criterion. Second, they determined which of these states should account for which of the three modes observed by Ionescu *et al.* [9]. The decision that a particular open or closed state should account for the mode showing a low, intermediate or high level of activity was based on heuristic inspection of the ligand-dependency of modal gating. The model was parametrized by optimizing a likelihood that accounted for various sources of single channel data including statistics of modal gating. This treats the parameter space of their model as a black box from which a suitable set of parameters capable of accounting for all datasets is selected by optimization. We expect such an approach to be statistically less efficient than a model whose structure incorporates modal gating more explicitly.

Siekmann *et al.* [12] used modal gating as the underlying construction principle of their model by separating the inference of parameters related to dynamics within modes from estimation of parameters related to switching between modes. First, models for the inactive mode M^1^ and the active mode M^2^ were inferred by fitting segments of data representative of each of the two modes—in fact, the same models were reused in the present study. However, because at that time rigorous statistical techniques for segmenting ion channel data by modes were not available, the time scales of the switching between both modes was inferred by connecting the submodels for M^1^ and M^2^ with a pair of transition rates whose values were then determined from a fit to complete traces of single channel data. Similar to Ullah *et al.* [11] modal gating was thus incorporated into the model without explicitly considering its stochastic dynamics apparent in the data.

In this study using our previously developed method Siekmann *et al.* [6], we were able to explicitly account for transitions between different modes inferred from experimental data. The method partitions a time series into segments based on the open probability *P*_*O*_ of the channel. In this way, spontaneous changes of channel activity can be detected. Because the analysis is based on Bayesian statistics, comprehensive information on the uncertainty of the results is available via the posterior distribution. For the IP_3_R data used here the inferred times at which the channel made a transition to another mode had very low estimated standard deviations (less than one data point up to a few data points). Whereas for this study, it was therefore sufficient to use point estimates of the change times, the full posterior distribution can be used for channels whose modes cannot be distinguished with similar accuracy.

Thus, the statistical method from Siekmann *et al.* [6] enables us to fit a model $\tilde{M}$ directly to the stochastic process of mode switching inferred from the experimental data instead of arbitrarily introducing transition rates between modes as in our previous study [12]. Therefore, we can accurately represent mode switching, only adding exactly as many parameters as required. In comparison with our previous model, the new model described here requires only two additional parameters. Inspection of the sojourn time histograms show that these two parameters are essential in order to account for the fact that sojourns in the nearly inactive mode M^1^ exhibit two different time scales which cannot be represented by a model with less parameters.

Because Siekmann *et al.* [6] distinguished modes based on their open probabilities, it may be difficult to distinguish modes with similar characteristic open probabilities *P*_*O*_ but with different kinetics, like, for example, an active mode with high *P*_*O*_ and a flicker mode with similiar *P*_*O*_ but faster transitions between open and closed states. This possibility can be excluded by fitting models *Q*^*i*^ to the observed open and closed events in segments representative for each mode. For the IP_3_R dataset used for this study we could not only show that *Q*^1^ and *Q*^2^, respectively, did not differ significantly for segments from the same time series but were also ligand-independent.

The hierarchical Markov model developed in this study allows us to combine the ‘modules’ *Q*^*i*^ accounting for opening and closing within modes with $\tilde{M}$ representing mode switching to a representation of both aspects of the single channel dynamics. It is important to note that none of the components $\left(({\tilde{m}}_{0},\tilde{M}),{\left(p^{i},Q^{i}\right)}_{i=1}^{n_{M}}\right)$ of our model were determined by directly fitting to the sequence of open and closed events observed in experiments—the models *Q*^*i*^ are inferred from segments of the data and the model $\tilde{M}$ is parametrized from transitions between the modes *M*^*i*^. Thus, the open and closed time distributions *f*_*O*_(*t*) and *f*_*C*_(*t*), respectively, can be considered a prediction of our hierarchical model *M*. That the hierarchical model *M* performs better at predicting *f*_*O*_(*t*) and *f*_*C*_(*t*) than our previous model whose transition rates were inferred from a direct fit to complete traces of open and closed events provides additional evidence that our new approach does not suffer from possible sources of error in our statistical analysis of modal gating but is, in fact, a superior representation of the data.

For analysing statistical properties of modal gating, an advantage of our model is that in addition to representing the channel being open or closed each state is also associated with a mode. The analysis of bursts according to Colquhoun & Hawkes [7] depends on the selection of open and ‘short-lived’ closed states that represent a burst and a class of ‘long-lived’ closed states that account for gaps between bursts. This not only requires additional assumptions but it is also unclear how the state space of an unstructured Markov model should be partitioned if a channel has multiple modes. No such difficulties arise in our model because each mode is represented by an aggregate of open and closed states. It is, therefore, clear how the relevant states should be chosen so that various properties such as, for example, the open and closed times of each mode, can be calculated using the theory of Colquhoun & Hawkes [2] or Colquhoun & Hawkes [7]. Because here, mode switching is defined as spontaneous changes between different open probabilities rather than clusters of open events, the methods from Colquhoun & Hawkes [2] seem more suitable. The theory of Colquhoun & Hawkes [7] leads to more complicated calculations due to the assumption that bursts must commence with an opening of the channel, whereas this does not necessarily have be the case for a sojourn in a mode. In summary, this means that an additional benefit of our modelling approach is that statistical properties of modes can be calculated more easily from our model than from a general Markov model.

The modular structure of our hierarchical model which separates the representation of transitions between modes (inter-modal kinetics) from the dynamics within modes (intra-modal kinetics) not only provides a more parsimonious representation than previous models but, most notably, evidence is accumulating that in channels that exhibit different modes the switching between modes may be more important for their physiological function than intra-modal kinetics. This is strongly suggested by recent studies of the IP_3_R. In a study of insect type I IP_3_R, Ionescu *et al.* [9] observed three modes with essentially identical kinetic properties across different ligand concentrations, whereas the overall dynamics of the channel was determined by the highly ligand-dependent prevalence of the channel in these modes. Thus, Ionescu *et al.* [9] proposed that modal gating is the major mechanism of ligand regulation in the IP_3_R. This was confirmed for mammalian type I and type II IP_3_R data by Siekmann *et al.* [6] and led to the interpretation that ion channel kinetics is restricted to a fixed repertoire of modes which have to be mixed appropriately in order to respond to given ligand concentrations. Ligand-dependent switching between ligand-independent modes suggests that physiological function may depend more strongly on the slow time scale of switching between modes rather than the fast opening and closing of the channel within a mode. This was indeed recently shown in two studies of the role of IP_3_R in intracellular calcium dynamics. Cao *et al.* [26] showed that the essential features of calcium oscillations in airway smooth muscle could be preserved after iteratively simplifying the model from Siekmann *et al.* [12] to a two-state model that only accounted for switching between the two modes neglecting the kinetics of transitions between multiple open and closed states within the modes. Siekmann *et al.* [27] applied similar reduction techniques to demonstrate that also the stochastic dynamics of small clusters of IP_3_R s can be captured by a two-state model reduced to the dynamics of mode switching. In our new hierarchical model, inter-modal and intra-modal kinetics are represented separately so that the model representation with the right level of detail can be chosen based on the requirements of a specific application.

### (b) Biophysical implications of modal gating

Although modal gating has been observed for a long time it has rarely been accounted for in ion channel models. The crucial importance of modal gating has only recently been appreciated among investigators of the IP_3_R channel and it is now widely recognized in the community [10]. Various independent sources of evidence indicate that modal gating must be accounted for, both for understanding IP_3_R function as well as for gaining insight into biophysical properties of the channel molecule. As mentioned in the previous section, the role of IP_3_R in intracellular calcium dynamics is defined by its behaviour on the slow time scale of transitions between different modes rather than the fast time scale of opening and closing [26,27]. Previously, Ionescu *et al.* [9] discovered that the IP_3_R adjusts its level of activity depending on ligands such as calcium by regulating the proportion of time that the channel spends in different modes. This was subsequently confirmed by the statistical analysis by Siekmann *et al.* [6]. These results reveal the major functional implications of modal gating, so one may ask if any insight can be gained into the underlying biophysics. In their early model of modal gating in a chloride channel, Blatz & Magleby [8] postulated that different modes may be related to different conformations of the channel protein. Direct experimental evidence into how different modes arise from biophysical constraints of the channel protein is accumulating. Two examples include a thorough analysis of the potassium channel KscA discussed in more detail below [28–30] and a more recent study by Vij *et al.* [31] on the acethylcoline receptor. Also see the commentary by Geng & Magleby [32]. This suggests that modes form a fixed repertoire of possible behaviours defined by the molecular properties of the channel. Being constrained to a few different modes, ion channels overcome these limitations by switching between modes.

This implies that methods for identifying different modes in single channel data not only provide us with more accurate insight into the channel dynamics but may also reveal the transitions between different biophysical states of the channel. As mentioned above there are strong indications that each mode is reflected by a different three-dimensional arrangement of the channel protein, known as a conformational state. Thus, the aggregates of states in ion channel models that account for different modes *M*^*i*^ correspond to different conformations at the level of the channel protein. In such a model, the transitions between states representing different modes reflect the rates of conformational changes.

This direct correspondence between aggregates of states and underlying biophysics is important to note because interpreting individual states in Markov models for ion channels is problematic in general, at least without additional experiments. For the simplest possible representation of a gating ion channel is a two-state Markov model with only one open and one closed state it is, of course, obvious that these two different model states at the same time correspond to different biophysical states of the channel protein. This ‘mechanistic’ interpretation explains the popularity of this type of model. The Markov assumption implies that the open and closed times of a two-state model are exponentially distributed which means that durations of channel openings and closings both have characteristic time scales *τ*_*O*_ and *τ*_*C*_ given by the parameters of the exponential sojourn time distributions *f*_*O*_(*t*) and *f*_*C*_(*t*). However, many ion channels exhibit multiple characteristic open and closed times that cannot be represented by exponential distributions. Whereas an open ion channel must be in a different conformation than a closed ion channel distinguishing only two conformational states is a very coarse description of the complicated deformations of channel proteins that can be identified by molecular dynamics models. Nevertheless, if our goal is to base our models on rigorous statistical analysis, for some data we may not be able to identify more than two states.

Non-exponential open and closed times can often be represented satisfactorily by aggregated continuous-time Markov models where more than one state is used for representing the channel being open or closed. These models provide a simple generalization of the two-state Markov model and account for more than just one characteristic open or closed time scale *τ*_*O*_ and *τ*_*C*_. By definition, the sojourn times in the open or closed class of an aggregated Markov model are distributed according to a phase-type distribution, a class of distributions representing the time a Markov chain spends in a set of transient states until exiting to an absorbing state [16,17]. As with the two-state model it is tempting to also associate the individual states of an aggregated Markov model with different biophysical states of the channel protein. The multiple open and closed states of an aggregated Markov model could be interpreted to resolve in more detail the series of conformational changes that the channel goes through while it opens. If this interpretation was valid one could hope to discover details of the molecular structure of ion channels beyond the trivial distinction between an open and a closed state once the ‘best’ aggregated Markov model for a given dataset has been found.

Unfortunately, this ‘mechanistic’ interpretation of aggregated Markov models has several flaws. Whereas it can be directly inferred from single channel data if the channel is open or closed and in which of its modes *M*^*i*^ it is, distinguishing different open or closed Markov states requires additional experiments and is possibly ill-defined. First, the only reason that a particular model consists of multiple open and closed states is that multiple characteristic open and closed times were observed. It is an assumption to be empirically confirmed that for each observed exponentially distributed sojourn time the channel must necessarily be in a distinct conformational states—so more Markov states may appear in the model than can be distinguished biophysically at the level of the channel protein. By contrast, it is likely that some conformational states may not have a strong enough influence on the dynamics that they are represented by a state in a model inferred from the data. But even if we assume that each Markov state should, in principle, reflect a distinct underlying biophysical state, it is challenging both experimentally as well as theoretically to identify, for example, a three-dimensional configuration of the channel protein that corresponds to a model state with a short open time and distinguish it from another conformational state that is characterized by a long open time.

Second, and more importantly, aggregated Markov models are only defined up to equivalence [20,33–36] with other models having the same number of open and closed states. In particular, it can be shown that models with completely different adjacency matrices can describe the same process [35] although there is a canonical phase-type description, given, for example, by its Laplace–Stieltjes transform. Thus, interpreting the graphical structure of an aggregated Markov model as a description of possible transitions between different conformational states is not necessarily meaningful without further data. A related problem is the fact that some adjacency matrices lead to non-identifiable models, in particular, certain types of cyclic models are non-identifiable. Whereas it is unlikely that transitions between conformational states underlie any fundamental restrictions of this kind, only some of these transitions would be identifiable from experimental data. It is important to note that the described challenge of relating aggregated Markov models with biophysical processes does not restrict in any way their capability of statistically capturing the stochastic dynamics of ion channels. This only demonstrates that aggregated Markov models are a more abstract representation than they may appear to be at first glance.

In summary, because it is much less problematic to associate aggregates of states with different underlying biophysical states than individual states within an aggregate, interpreting mode switching as transitions between distinct biophysical states does not suffer from these difficulties. Chakrapani *et al.* [28–30] were able to restrict the KscA channel to one of its normally four modes by mutating a particular site of the amino acid sequence of the channel protein. Combining crystallography imaging and molecular dynamics modelling they could further demonstrate that the four modes were related to different conformational states of the channel. It is therefore likely that switching between distinct characteristic dynamical patterns in single channel data can be directly associated with the transition from one to another conformation of the channel protein. This implies that models which accurately represent mode switching can also be used to infer the time scales of transitions between biophysical states associated with these modes. This opens up the exciting possibility that we can gain insight into biophysical processes involved in ion channel gating by statistical analysis and modelling of single channel data rather than having to rely on more time-consuming experimental techniques such as crystallography or more laborious modelling techniques such as molecular dynamics.

## Data accessibility

Data and code used for this study have been published on github. The most current version can be obtained from https://github.com/merlinthemagician/icmcstat.

## Appendix A. Mathematical background

The results presented in the main text are derived from the following properties of the Kronecker product and sum and some well-known results from linear algebra.

Proposition A.1 (Properties of Kronecker product ⊗ and Kronecker sum ⊕) *The following properties of the Kronecker product and sums can all be found in Horn & Johnson* [37].

(*i*) *Transposition and conjugate transpose* (*Properties* 4.2.4 *and* 4.2.5):
  A 1 $${\left(A⊗B\right)}^{T}=A^{T}⊗B^{T},\;{\left(A⊗B\right)}^{*}=A^{*}⊗B^{*}.$$
(*ii*) *Compatibility of tensor product and matrix multiplication* (*Lemma* 4.2.10): *Let* $A\in R^{k_{1}\times m_{1}},$ $C\in R^{m_{1}\times n_{1}},$ $B\in R^{k_{2}\times m_{2}},$ $D\in R^{m_{2}\times n_{2}}$.
  A 2 $$(A⊗B)(C⊗D)=(AC)⊗(BD)\in R^{k_{1}k_{2}\times n_{1}n_{2}}.$$
(*iii*) *Eigenvalues of Kronecker sums* *A*⊕*B* (*Theorem* 4.4.5): *Let* *α*, *β* *denote eigenvalues of the square matrices* *A* *and* *B*. *Then the eigenvalues of* *M*=*A*⊕*B* *are*
  A 3 γ=α+β.
(*iv*) *Matrix exponentials of Kronecker sums* (*ch. 6, Problem 14*): *For square matrices* $A\in R^{m\times m}$ and $B\in R^{n\times n}:$
  A 4 $$\exp(A⊕B)=\exp(A)⊗\exp(B)\in R^{mn\times mn}.$$

If we cannot assume that a matrix has a complete set of eigenvectors so that it may not be diagonalizable we can still triangularize this matrix over the complex numbers C. The process of triangulation can be described by the Schur decomposition.

Proposition A.2 (Schur decomposition) *For a square matrix* $A\in R^{m\times m}$ *there exists a unitary matrix* $\Theta \in C^{m\times m}$ *and an upper triangular matrix* *T* *such that*

A 5 $$T=\Theta^{*}A\Theta ,$$

*where* *Θ** *is the conjugate transpose of* *Θ*; (*A 5*) *is known as the Schur decomposition*. *Let* $A\in R^{m\times m}$ *and* $B\in R^{n\times n}$ *with Schur decompositions*

$$T_{A}=V^{*}AV\;\mathrm{and}\;T_{B}=W^{*}BW.$$

*Schur decompositions for the Kronecker product* *A*⊗*B* *and the Kronecker sum* *A*⊕*B* *can then be obtained via*

A 6 $$T_{A⊗B}={\left(V⊗W\right)}^{*}A⊗B(V⊗W),\;T_{A⊕B}={\left(V⊗W\right)}^{*}A⊕B(V⊗W).$$

Proof. See Horn & Johnson [38], theorem 2.3.1. For (A.6), we refer to the proofs of Theorems 4.2.12 and 4.4.5 in [37]. ▪
